# Identifying rare disease variants in the Genetic Analysis Workshop 17 simulated data: a comparison of several statistical approaches

**DOI:** 10.1186/1753-6561-5-S9-S17

**Published:** 2011-11-29

**Authors:** Ruixue Fan, Chien-Hsun Huang, Shaw-Hwa Lo, Tian Zheng, Iuliana Ionita-Laza

**Affiliations:** 1 Department of Statistics, Columbia University, 1255 Amsterdam Avenue, MC 4690, New York, NY 10027, USA; 2 Department of Biostatistics, Mailman School of Public Health, Columbia University, 722 West 168th Street, New York, NY 10032, USA

## Abstract

Genome-wide association studies have been successful at identifying common disease variants associated with complex diseases, but the common variants identified have small effect sizes and account for only a small fraction of the estimated heritability for common diseases. Theoretical and empirical studies suggest that rare variants, which are much less frequent in populations and are poorly captured by single-nucleotide polymorphism chips, could play a significant role in complex diseases. Several new statistical methods have been developed for the analysis of rare variants, for example, the combined multivariate and collapsing method, the weighted-sum method and a replication-based method. Here, we apply and compare these methods to the simulated data sets of Genetic Analysis Workshop 17 and thereby explore the contribution of rare variants to disease risk. In addition, we investigate the usefulness of extreme phenotypes in identifying rare risk variants when dealing with quantitative traits. Finally, we perform a pathway analysis and show the importance of the vascular endothelial growth factor pathway in explaining different phenotypes.

## Background

In disease association studies, the common disease/common variants (CDCV) model states that common diseases are caused by common variants with minor allele frequencies (MAFs) in the range of 1–5%. Recent studies suggest that although many more common disease susceptibility variants may still exist, they will likely have even smaller effect sizes and thus will be unlikely to explain most of the missing heritability for many of the traits [1]. On the other hand, the common disease/rare variants (CDRV) hypothesis assumes that complex disorders are caused by multiple rare variants (with MAF < 1% or 5%), most of which are missense mutations that can alter gene expression level or change amino acid sequences directly [2,3]. Therefore the detection and investigation of rare variants will help researchers further understand the disease etiology and may provide new insights into medical treatments. With the development of next-generation sequencing technologies, large numbers of single-nucleotide polymorphisms (SNPs) with low frequencies can be detected in a relatively short time and at relatively low cost [4], making the association study with rare variants increasingly feasible.

Many statistical methods have been developed to test association between common SNPs and disease traits, but these methods have low power for identifying rare variants in complex diseases because of the low frequencies and large number of such variants [5,6]. Several new methods have been developed that analyze all rare variants in a gene or a candidate region. Li and Leal [7] proposed one of the first statistical methods for the analysis of rare variants; their method is based on testing whether the proportion of carriers of rare variants is significantly different between case and control groups. A subsequent paper by Madsen and Browning [8] introduced the concept of weighting variants according to their estimated frequencies in control subjects so that less frequent variants are given higher weight compared with more common variants. Price et al. [9] extended the weighted-sum approach of Madsen and Browning to weight variants according to externally defined weights, such as predictions about the probability of a variant to be functional. Ionita-Laza et al. [10] developed a new approach, also based on a weighted-sum statistic, that can be more powerful than the other methods mentioned, especially in larger genetic regions and in cases where a mixture of risk and protective variants is present in the region of interest.

In this paper, we apply these methods to the simulated data sets provided by Genetic Analysis Workshop 17 (GAW17) to analyze the effect of rare variants on various phenotypes and evaluate the performance of each method by comparing the results with the true disease model. We also study the effect of trait dichotomization when dealing with quantitative traits. In addition, we investigate the importance of the vascular endothelial growth factor (VEGF) pathway to different phenotypes in the GAW17 data set.

## Methods

### Data set

The simulated data set from GAW17 consists of 3,205 autosomal genes with 24,487 SNPs genotyped on 697 subjects. Three quantitative phenotypes (Q1, Q2, Q4) and the Affected Status phenotype are generated for each of the unrelated individuals, and 200 simulated replicates are available to us. In particular, each simulation contains 209 case subjects and 488 control subjects for the Affected Status phenotype and three normally distributed quantitative traits (Q1, Q2, Q4). In the disease model, Q1 is caused by SNPs in the VEGF pathway and Q2 is caused by SNPs related to cardiovascular risk and inflammation; the trait Affected Status is affected by SNPs from both biological processes. See Blangero et al. [11] for more details on the simulation model.

### Statistical testing strategies

We implemented three recently developed strategies for association testing with rare variants: the gene-based combined multivariate and collapsing (CMC) method of Li and Leal [7], the weighted-sum (WS) method of Madsen and Browning [8], and the replication-based (RB) approach of Ionita-Laza [10] in our own R software package. Briefly, the CMC method is used to test the difference in the proportion of rare variant carriers between case and control subjects. The WS method assigns higher weight to variants less frequent in control subjects and obtains a weighted-sum score from case subjects. The RB approach is based on partitioning observed variants into two disjoint classes (likely to be risk or protective) and uses a weighting scheme that reflects the difference in observed frequencies between case and control subjects. See Dering et al. [12] for more descriptions of the CMC and WS methods; the RB approach is described in full detail by Ionita-Laza et al. [10].

### Strategies for quantitative traits

Because the methods mentioned are designed for case-control studies, we make some adjustments in order to apply them to quantitative traits. First, we dichotomize the quantitative traits by treating the individuals in the upper quartile as case subjects and those in the lower quartile as control subjects and then apply the CMC, WS, and RB methods to these pseudo-case-control data. Another strategy that uses all the available data is to use the modified weighted-sum test suggested by Price et al. [9] in which each SNP’s weight is calculated on the basis of its frequency in all subjects.

### Pathway analysis

The true disease model suggests that Q1 and Affected Status are affected by genes from the VEGF pathway [11]; therefore we collapsed all the rare variants in the VEGF pathway and performed a pathway-based analysis. We searched the VEGF signaling pathway in the KEGG database (http://www.genome.jp/kegg/pathway.html) and in the NCI-pathway interaction database (http://pid.nci.nih.gov/) and found that 53 genes in GAW17 data sets participate in the VEGF pathway. We grouped the 344 rare SNPs (MAF < 0.05, with 173 nonsynonymous SNPs) from these 53 genes and evaluated their effects on Affected Status and Q1.

## Results and discussion

### Prominence of rare variants

A preliminary analysis indicates that the distribution of SNP frequencies is heavily skewed toward low frequencies. In particular, about 87% of SNPs have a MAF less than 0.05, and about 74% have a MAF less than 0.01. Motivated by the large proportion of rare variants, we decided to investigate their effects on different phenotypes even without prior knowledge of the true disease model.

We applied the gene-based CMC, WS, and RB methods for the case-control design and dichotomized quantitative traits; we used Price’s method for quantitative traits only. For each gene we computed the *p*-value and the relative rank of the gene among all genes in each method, using only the first simulated data set. We also obtained the power (or replicability) for each gene, calculated as the percentage of times the resulting gene-based *p*-value was less than 0.05 across the 200 replicates. After the true disease model was reviewed in GAW17, our results were compared with the true model. In Table 1 we report results for the known causal genes that rank in the top 50 with any of the methods.

**Table 1:** *p*-values and ranks of disease genes among all genes using the first simulated data set and their power estimates across 200 simulations

Phenotype	Gene	*p*-values and ranks				Power
			
		WS	CMC	RB	Price et al. [9] (Q-traits only)	
Affected status	*FLT1*	9.0 × 10^−4^/1.9 × 10^−3^ (9.5/25)	2.1 × 10^−5^/4.3 × 10^−3^ (2/46)	8.0 × 10^−4^/3.0 × 10^−3^ (8/29)	–	0.78/0.685
	*VEGFA*	3.3 × 10^−3^/1.4 × 10^−3^ (16/19)	3.6 × 10^−3^/2.2 × 10^−3^ (22/25)	3.0 × 10^−3^/2.0 × 10^−3^ (22/20)	–	0.06/0.06
	*PIK3C2B*	7.1 × 10^−3^/6.1 × 10^−3^ (33/45.5)	7.0 × 10^−3^/9.3 × 10^−3^ (39/75)	1.0 × 10^−2^/7.0 × 10^−3^ (43/40)	–	0.64/0.71

Q1	*FLT1*	<1.0 × 10^−4^/<1.0 × 10^−4^ (1.5/1.5)	4.2 × 10^−11^/1.3 × 10^−5^ (1/3)	<1.0 × 10^−4^/<1.0 × 10^−4^ (1.5/1.5)	<1.0 × 10^−7^/<1.0 × 10^−7^ (1/1)	1/1
	*KDR*	5.0 × 10^−4^/3.0 × 10^−4^ (6/10)	5.3 × 10^−4^/3.0 × 10^−4^ (8/9)	7.0 × 10^−4^/4.0 × 10^−4^ (7/7)	1.2 × 10^−6^/1.8 × 10^−7^ (2/2)	0.995/0.99
	*HIF1A*	9.8 × 10^−3^/9.8 × 10^−3^ (41/89.5)	1.0 × 10^−2^/1.0 × 10^−2^ (40.5/96)	2.4 × 10^−2^/2.1 × 10^−2^ (45/104)	1.1 × 10^−2^/2.1 × 10^−2^ (96/256)	0.655/0.61
	*VEGFA*	1.4 × 10^−2^/1.9 × 10^−3^ (51/28)	1.5 × 10^−2^/5.0 × 10^−4^ (49.5/13)	3.0 × 10^−2^/3.4 × 10^−3^ (59/27)	2.2 × 10^−3^/5.6 × 10^−4^ (22/33)	0.015/0.485
	*ARNT*	1.6 × 10^−2^/9.0 × 10^−4^ (56/19)	1.7 × 10^−2^/1.5 × 10^−3^ (63/24)	2.2 × 10^−2^/1.9 × 10^−3^ (44/18.5)	2.0 × 10^−2^/5.9 × 10^−3^ (156/128)	0.845/0.88
	*VEGFC*	0.5/0.5 (956/1521)	0.502/0.502 (913/1313)	1/1 (1460/2083)	1.4 × 10^−3^/1.4 × 10^−3^ (19/63)	0.355/0.345

Q2	*VNN3*	8 × 10^−4^/0.003 (2/7)	1.2 × 10^−2^/2.2 × 10^−2^ (9/40)	2.4 × 10^−3^/7.0 × 10^−3^ (5/12)	1.4 × 10^−3^/2.4 × 10^−3^ (6/19.5)	0.545/0.485
	*PDGFD*	1.8 × 10^−2^/3.6 × 10^−2^ (30/113)	4.1 × 10^−2^/6.5 × 10^−2^ (46.5/118)	2.6 × 10^−2^/4.5 × 10^−2^ (27/79)	4.4 × 10^−2^/5.2 × 10^−2^ (238/420)	0.545/0.485
	*PLAT*	1.6 × 10^−2^/3.2 × 10^−1^ (24/975)	6.3 × 10^−2^/4.2 × 10^−1^ (66/1030)	3.9 × 10^−2^/9.2 × 10^−1^ (46/1952)	1.2 × 10^−1^/4.9 × 10^−1^ (452/1960)	0.255/0.075
	*LPL*	2.3 × 10^−2^/4.1 × 10^−2^ (42/124)	6.5 × 10^−2^/8.1 × 10^−2^ (70/172.5)	3.4 × 10^−2^/9.8 × 10^−2^ (40/186)	8.2 × 10^−2^/2.2 × 10^−1^ (1999/1166)	0.47/0.28
	*VNN1*	1.2 × 10^−1^/2.2 × 10^−1^ (176/629)	1.6 × 10^−1^/3.8 × 10^−1^ (142/957)	2.2 × 10^−1^/3.3 × 10^−1^ (242/624)	1.9 × 10^−3^/3.2 × 10^−1^ (11/1468)	0.38/0.04

In each cell the top line lists the *p*-values and the values in parentheses give the corresponding ranks. Results are computed using nonsynonymous rare variants (before slash) or all rare variants (after slash). Power is the replicability across 200 simulations using the WS method. Genes are sorted by their *p*-values in the WS method using nonsynonymous rare variants.

### Effect of minor allele frequency on power

In our analyses, we set two different thresholds to define rare variants: MAF < 0.01 (T1) or MAF < 0.05 (T5). We note here that the results from T1 and T5 are consistent but using T5 tends to give higher power compared to T1 because the sample size (697) is relatively small and the disease model involves variants with MAF greater than 0.01. Therefore, for the sake of clarity, we report here and in Table 1 only the results from using cutoff T5. In this data set, there are 21,355 rare SNPs with MAF < 0.05, and 12,193 of them are nonsynonymous SNPs, which we analyzed using various approaches.

### Disease genes identified in the first simulation

We observe that for case-control or dichotomized quantitative trait designs, *p*-values and their ranks from the WS, CMC, and RB methods highly agree with each other; this makes us confident about the true effect if we observe strong signals across different methods. Our analyses included either all the genes (2,874 in total) harboring rare variants or only those genes (1,999 in total) containing nonsynonymous rare variants. Among these genes, *FLT1* is the easiest to identify. For Q1, *FLT1* is statistically significant with *p*-values less than the Bonferroni-corrected significance level (~1.67 × 10^−5^) and ranks first among all genes in the first simulation. For Affected Status, *FLT1* has a very low *p*-value, close to the Bonferroni-corrected threshold, especially when only nonsynonymous rare mutations are used. For Q2, disease gene *VNN3* is top-ranked with all three methods.

For quantitative traits, the two strategies (dichotomization or not) result in different risk genes being detected; in particular, dichotomization identifies more genes with higher power compared with Price’s method. This is partly because dichotomization likely eliminates rare disease variants contained only in individuals with modest phenotype values but it amplifies the signal for rare variants with strong effect that show up in extreme phenotypes. Genes identified by dichotomization tend to have high power because the statistical signal can be consistently detected after removing unidentifiable noise. Therefore selecting extreme phenotypes can sometimes be an alternative strategy in dealing with quantitative traits. On the other hand, these two strategies both detect the same disease genes: *FLT1* and *KDR* for Q1 and *VNN3* for Q2, which may suggest that they are true positives.

Overall, our results are encouraging on these simulated data. In particular, we successfully detected six true disease genes for Q1, five for Q2, and three for Affected Status in the top 50 most significant genes using only the first simulated data set.

### Replicability across 200 simulations

Many of the disease genes are highly replicable in 200 simulations with power greater than 50% (Table 1), indicating a potentially true signal. However, there are numerous consistent false-positive genes showing high replicability. Some top genes identified in the first simulation, such as *GOLGA1* for Affected Status, *PPP1R14BP1* for Q1, and *MAP3K8* for Q2, all have power greater than 50% (data not shown). This can happen for various reasons. Both sequencing errors and population stratification can create these replicable false signals, especially in these data sets, where the genotype data are fixed across simulations. Another reason might be long-range correlation between markers. Our investigation showed that certain consistent false positives are highly correlated with true causal SNPs and that it is difficult to eliminate this artificial linkage effect because of the fixed-genotype design across all simulations.

### Filtering variants based on known functional predictions

We compared the results using only nonsynonymous rare variants with those using all rare variants and observed that nonsynonymous rare variants improve the rank of true disease genes and the power for most disease genes, especially for trait Q2 (Table 1). This is because the true disease model includes only nonsynonymous mutations. This may hold true for real data as well because most rare variants are missense mutations and can change gene expression level or protein function directly. Hence focusing on nonsynonymous mutations helps to increase signal-to-noise ratio for real risk factors.

### Pathway analysis

We collapsed the rare SNPs (MAF < 0.05) in the VEGF pathway and evaluated their effects on traits Affected Status and Q1 using different statistical strategies (Table 2). The WS and RB methods performed substantially better than the CMC method, probably because of the increased power of weighted-sum approaches for large genomic regions. The CMC approach has decreased power as the region size increases because more individuals will be carriers of rare mutations by chance. Also, using nonsynonymous variants results in a lower *p*-value than using all variants does; this is consistent with previous gene-based analyses and emphasizes again that filtering nonsynonymous mutations is important for identifying influential rare SNPs.

**Table 2:** *p*-value of the VEGF pathway for Affected Status and Q1 in the first simulation using different methods

	Affected Status			Q1, dichotomized			Q1 (Price et al. [9])
		
	CMC	WS	RB	CMC	WS	RB	
Rare NS	0.00287	<0.0001	<0.0001	2.938 × 10^−6^	<0.0001	<0.0001	<0.0001
Rare	0.06721	0.02	0.0003	0.02687	<0.0001	<0.0001	<0.0001

Results from using only nonsynonymous rare variants (Rare NS) and all rare variants (Rare) are shown here. *p*-values for the WS and RB methods are computed based on 10,000 permutations.

## Conclusions

In this study, we applied three statistical methods to detect rare variants and successfully pinpointed several true disease genes containing rare SNPs in the GAW17 data sets. We started with the first data set and then made use of the remaining 199 replicates to evaluate the replicability of the discoveries in the first data set. This strategy agrees with the natural procedure when dealing with real data; namely, a first data set is used for discovery purposes, and additional independent data sets are used for replication purposes. We showed that using 5% as the MAF cutoff is better than using 1% in the GAW17 data set because the current sample size is small and disease variants occur at higher frequencies as well. Also, including only nonsynonymous mutations can substantially increase the signal. We also showed that selection of extreme phenotypic individuals can be a useful strategy for rare variant analysis with quantitative traits. Our analyses encountered the difficulty of numerous false positives, some probably resulting from sequencing errors or population stratification. Nevertheless, in real life without knowledge of the true model, the methods we investigated here can help us to detect rare disease SNPs. The consistency of results from different methods can be an indicator of true signal. The three methods performed similarly for the disease genes in these simulations. However, we did observe that the replication-based approach was the most powerful approach for large genetic regions such as pathways, followed by the weighted-sum and collapsing approaches.

## Competing interests

The authors declare that they have no competing interests.

## Authors’ contributions

II and RF designed the study. II, RF, SHL and TZ performed the research. II, RF and CHH analyzed data. II and RF wrote the paper. All authors read and approved the final manuscript.
